# The consequences of ignoring measurement invariance for path coefficients in structural equation models

**DOI:** 10.3389/fpsyg.2014.00980

**Published:** 2014-09-17

**Authors:** Nigel Guenole, Anna Brown

**Affiliations:** ^1^IBM Smarter Workforce InstituteLondon, UK; ^2^Institute of Management Studies, Goldsmiths, University of LondonLondon, UK; ^3^School of Psychology, Keynes College, University of KentCanterbury, UK

**Keywords:** measurement equivalence/invariance, categorical indicators, structural equation modeling

## Abstract

We report a Monte Carlo study examining the effects of two strategies for handling measurement non-invariance – modeling and ignoring non-invariant items – on structural regression coefficients between latent variables measured with item response theory models for categorical indicators. These strategies were examined across four levels and three types of non-invariance – non-invariant loadings, non-invariant thresholds, and combined non-invariance on loadings and thresholds – in simple, partial, mediated and moderated regression models where the non-invariant latent variable occupied predictor, mediator, and criterion positions in the structural regression models. When non-invariance is ignored in the latent predictor, the focal group regression parameters are biased in the opposite direction to the difference in loadings and thresholds relative to the referent group (i.e., lower loadings and thresholds for the focal group lead to overestimated regression parameters). With criterion non-invariance, the focal group regression parameters are biased in the same direction as the difference in loadings and thresholds relative to the referent group. While unacceptable levels of parameter bias were confined to the focal group, bias occurred at considerably lower levels of ignored non-invariance than was previously recognized in referent and focal groups.

## INTRODUCTION

Methodologists in the social sciences differentiate between two primary forms of psychometric equivalence, or ways of showing that a psychometric instrument functions similarly in a probabilistic sense across multiple populations. Measurement invariance exists when two individuals sampled from different sub-populations but with the same standing on the latent continuum have the same expected test score (Drasgow, 1982, 1984; Mellenbergh, 1989; Meredith, 1993; Vandenberg and Lance, 2000; Kankaraš et al., 2011; Davidov et al., 2014). Today measurement invariance is considered a fundamental issue in psychological testing (Lubke et al., 2003) that has social as well as statistical consequences (Beuckelaer et al., 2007; Borsboom et al., 2008). In studies of measurement invariance, the groups under study are designated as either the referent or focal group (Holland and Thayer, 1988). Next, the equivalence of measurement model parameters, usually the item loadings and intercepts or thresholds, is examined using approaches based on either item response theory (IRT) or multiple group confirmatory factor analysis (CFA). In the CFA approach, which is the focus of the present article, a series of competing models is fitted to response data, where the group membership acts as a potential categorical moderator (e.g., French and Finch, 2011). Equivalent measurement model parameters across groups are required for comparable measurement, a consideration identical to the use of equal measurement scales (say, degrees centigrade) when comparing temperatures in two different regions. For a recent description of the process of examining measurement invariance see French and Finch (2011) or van de Schoot et al. (2012).

Relational invariance, the second form of equivalence, examines whether the same structural relationships hold between variables across two or more subpopulations (Mellenbergh, 1989; Meredith, 1993). When variables under study are latent, the slopes of structural regression paths in multiple group analyses are examined for invariance ^1^. Drasgow (1984) has argued that there is a logical sequence to testing measurement equivalence: measurement invariance should first be tested, followed by relational invariance. If non-invariance is observed in the measurement model, the researcher might want to delete “offending” items. This might not be appropriate if the questionnaire is a well-established instrument. Remaining options include freely estimating the parameters for the non-invariant items to achieve partial invariance (Byrne et al., 1989), or to ignore the non-invariance. The challenge faced by the researcher who allows partial invariance is how much non-invariance can be tolerated whilst still claiming that the same construct is measured across groups or between current and past research. The challenge faced by the researcher ignoring the non- invariance is whether the results of the misspecified model can be trusted. In practice, applied researchers should make a decision based on the expected threats to the validity of their conclusions under each course of action.

Sometimes, the primary focus of the researcher is to examine structural relations across groups of interest. Wasti et al. (2000) for example, examined whether the antecedents and consequences of sexual harassment were the same between the United States and Turkey. If it was certain that ignoring measurement non-invariance across populations would lead to negligible differences in relationships between latent variables, it could be tempting to do so. On the other hand, it would be necessary to model the non-invariance if ignoring it would result in a substantial regression parameter bias. There have been at least three calls for Monte Carlo studies of such issues in the literature (Chen, 2008; Schmitt and Kuljanin, 2008; Schmitt et al., 2011). The present article addresses this call for a Monte Carlo study of measures employing categorical indicators. Our approach broadly follows the recommendations of Paxton et al. (2001) and Boomsma (2013).

### PAST RESEARCH ON THE EFFECT OF MEASUREMENT INVARIANCE ON RELATIONAL INVARIANCE

Chen (2008) reported a Monte Carlo investigation of the impact of ignoring measurement non-invariance in the slopes of linear factor models on the relative bias in regression parameters of structural models. She found that when referent group loadings were higher on the exogenous latent variable, the referent group regression parameter was overestimated, i.e., the relative bias was positive, and the regression parameter in the focal group was underestimated, i.e., the relative bias was negative. The pattern was reversed when the non-invariant construct was the latent criterion variable. The relative bias in the regression parameters was always greater in the focal group. However, extreme levels of non-invariance had to be ignored before adverse effects on regression coefficient accuracy emerged.

Oberski (2014) used Monte Carlo studies to examine the expected change in the parameter of interest statistic (EPC-Interest: Satorra, 1989; Bentler and Chou, 1992) as a method for examining the sensitivity of parameters under study to misspecification of invariance constraints. This method has the advantage of avoiding the unnecessary rejection of the measurement invariance model, and alerting the researcher to doubtful substantive conclusions about parameters when measurement invariance appears to hold. Unlike the more familiar expected parameter change (EPC: Saris et al., 1987), EPC-Interest examines the change in parameters of interest other than the parameter being fixed or freed. Obserski examined changes in regression parameters of a random effects model due to ignoring versus modeling non-invariant loadings. The effects on the regression coefficient in the empirical example used in that article were generally small.

### THEORETICALLY DERIVED RESEARCH QUESTION

We extend the work of Chen (2008) and Oberski (2014) in several new directions. While Oberski (2014) evaluated a method for examining the impact of the non-invariance problem in specific models, this study examines the extent of these effects in general structural relationships under typical conditions. Whereas Chen examined the impact of measurement non-invariance on simple regression parameters, structural models in practice are usually more complex. We examined the effect of ignoring non-invariance on partial regression coefficients, i.e., regression with covariates, mediated regression coefficients, and moderated regression coefficients. In each case, we examined the effect of ignoring the invariance when the latent variable with non-invariant parameters was the predictor, when it was the criterion, and when the latent variable with non-invariance occupied the mediator position in the model.

The current investigation extends the work of Chen (2008) and Oberski (2014) in a further important way. We generalize these authors’ earlier results to models incorporating categorical factor indicators, thus focusing on loadings and thresholds in categorical item factor analyses (CIFA: Forero and Maydeu-Olivares, 2009) rather than linear factor analyses. This meets the call of Chen who stated “one direction in future research is to systematically examine bias under various levels of invariance for categorical variables” (p. 1017).

### HYPOTHESES

The primary objective of this study is to examine the impact of misspecified measurement parameters on structural relations in commonly used regression models. The impact is expected to depend on the role that the latent factor with non-invariant measurement part plays in the model – whether it is an independent or a dependent variable in structural relationships. The secondary objective is to examine whether the patterns of results for either role are similar across simple regression, regression with covariates, moderated regression, and mediation models. Based on previous research (e.g., Chen, 2008; Oberski, 2014), we hypothesize the following basic effects pertaining to misspecified factors.

#### Loading parameters

When factor loadings in the focal group are lower than in the referent group, and this is ignored, the variance of the latent factor in the focal group will be underestimated. The net effect will be an overestimation of the regression coefficient in the focal group when the mis-specified factor is the latent *X*-variable (independent, or predictor variable) in the structural model. Conversely, the net effect is an underestimation of the regression coefficient in the focal group when the misspecified factor is the latent *Y*-variable (dependent, or criterion variable). The effects will be reversed for the referent group.

#### Threshold parameters

When item thresholds in the focal group are lower (i.e., an acquiescent response style exists in the focal group: Cheung and Rensvold, 2000), and this is ignored, the latent factor mean in the focal group will be overestimated. While the effect on the mean is the strongest expected effect of the distorted factor metric, a distortion to the latent factor variance in the focal group is also expected, with the variance underestimated in the focal group. The net effect will be an overestimation of the regression coefficient in the focal group when the misspecified factor is the latent *X*-variable (independent, or predictor variable) in the structural model. Conversely, the net effect will be an underestimation of the regression coefficient in the focal group when the misspecified factor is the *Y*-variable (dependent, or criterion variable). The effects are reversed for the referent group.

#### Loading and threshold non-invariance

When item thresholds are lower in the focal group (i.e., acquiescence is present) and factor loadings are also lower in the focal group the bias is expected be accentuated.

### EXPERIMENTAL CONDITIONS

#### Modeling approach

Two approaches were compared; namely, (1) modeling measurement non-invariance, whereby non-invariant item parameters across groups were freely estimated, and (2) ignoring measurement non-invariance, whereby non-invariant item parameters were constrained equal across groups. Variances / residual variances of the latent factors were set to 1 in the referent group, and freely estimated in the focal group. Structural regression parameters were freely estimated in both groups.

#### Type of measurement non-invariance studied

Three types of item non-invariance were considered in the study. First, we examined the effect of factor loading (a.k.a. metric) non-invariance. Second, we examined the effect of threshold (a.k.a. scalar or strict) non-invariance in the form of an acquiescent response style. Finally, we considered the simultaneous effect of both the loadings and thresholds non-invariance.

#### Types of structural model

**FIGURE 1:**
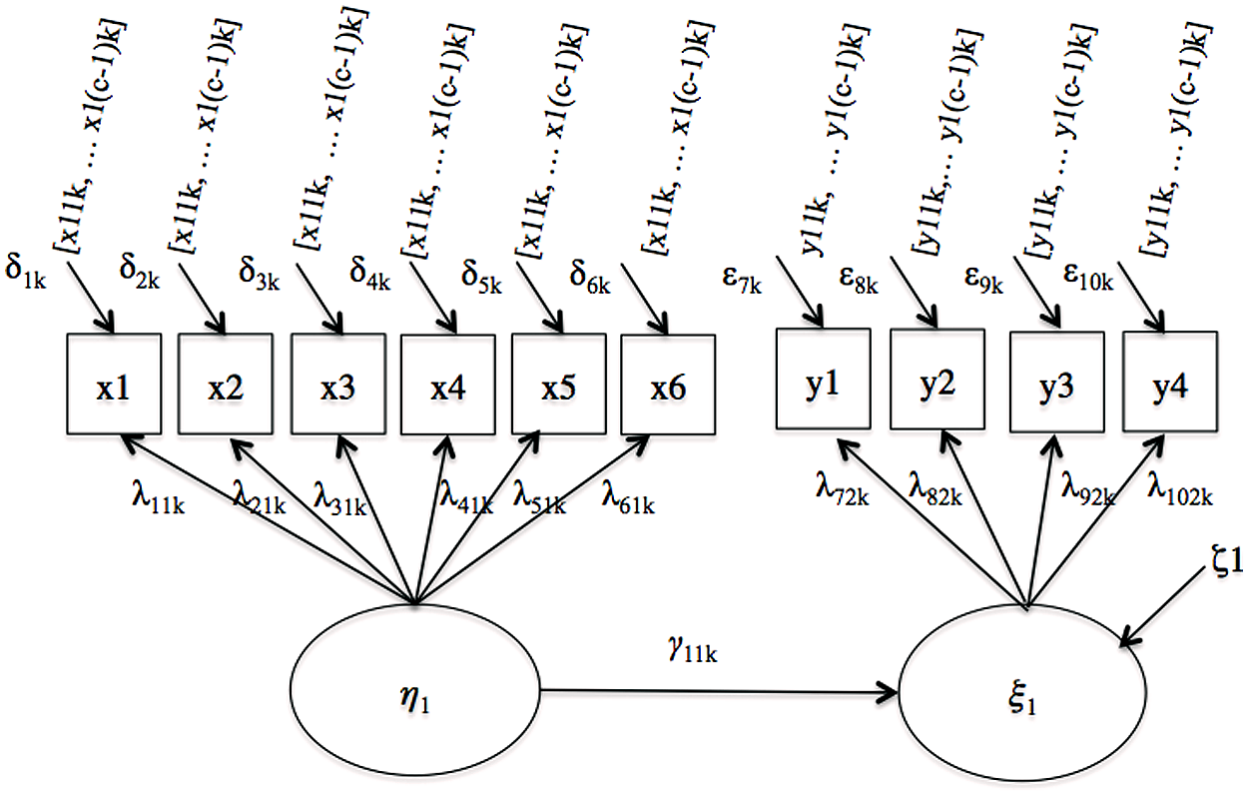
**Simple regression**.

The first model considered is a simple regression model illustrated in **Figure 1**. Two regression coefficients, γ_11,1_ and γ_11,2_, quantify the paths linking the latent predictor variable to the latent criterion variable in the referent and the focal group, respectively.

**FIGURE 2:**
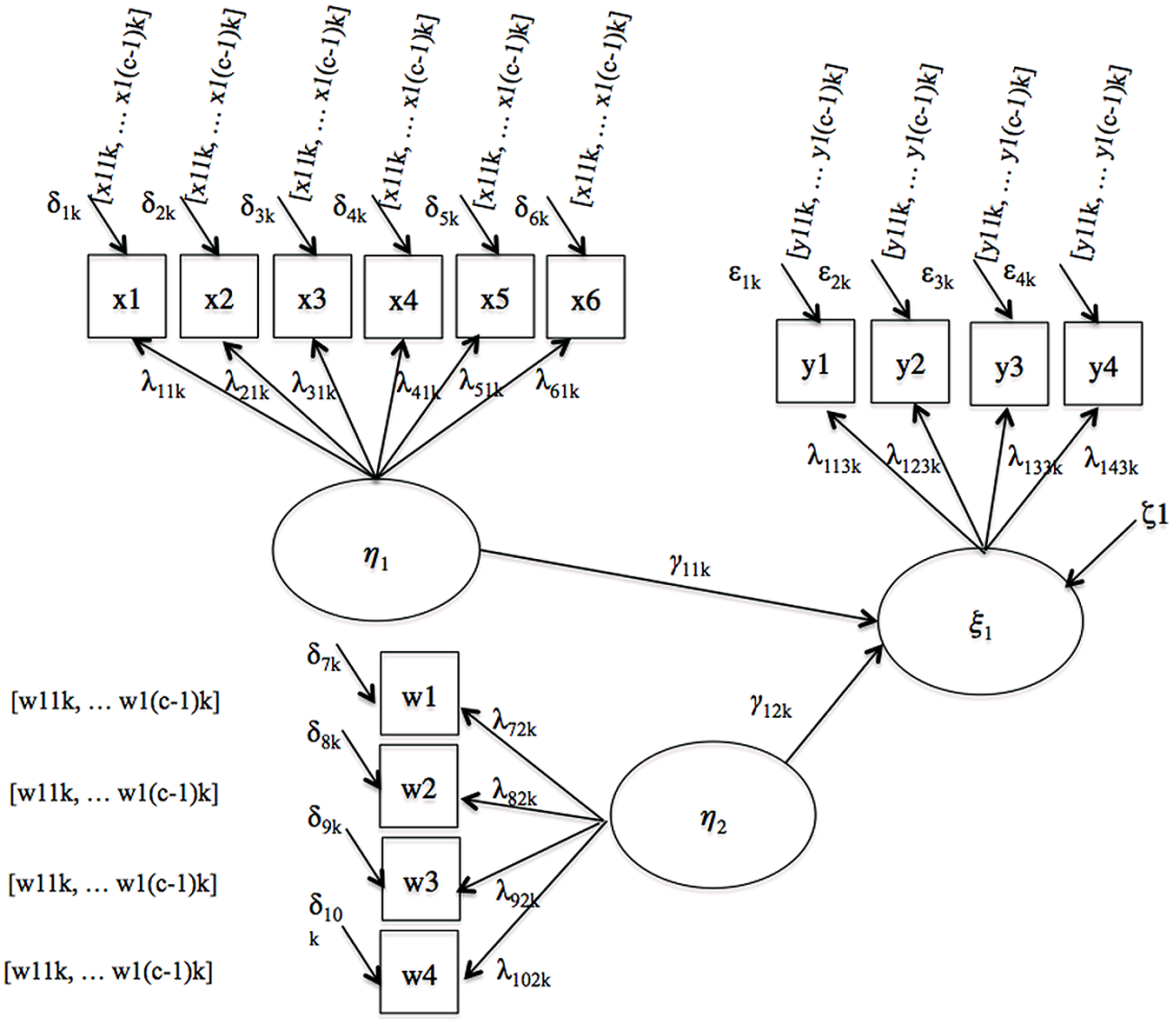
**Partial regression**.

Second, we considered a multiple regression model illustrated in **Figure 2**. In each group, the first regression coefficient represents the relationship between the predictor (target of our analysis) and the criterion, referred to as γ_11,1_ and γ_11,2_ in the referent and the focal group, respectively, while the second coefficient represents the relationship between the covariate and the criterion, referred to as γ_12,1_ and γ_12,2_. The population covariance of the predictors was fixed at zero, as it was not expected to impact results.

**FIGURE 3:**
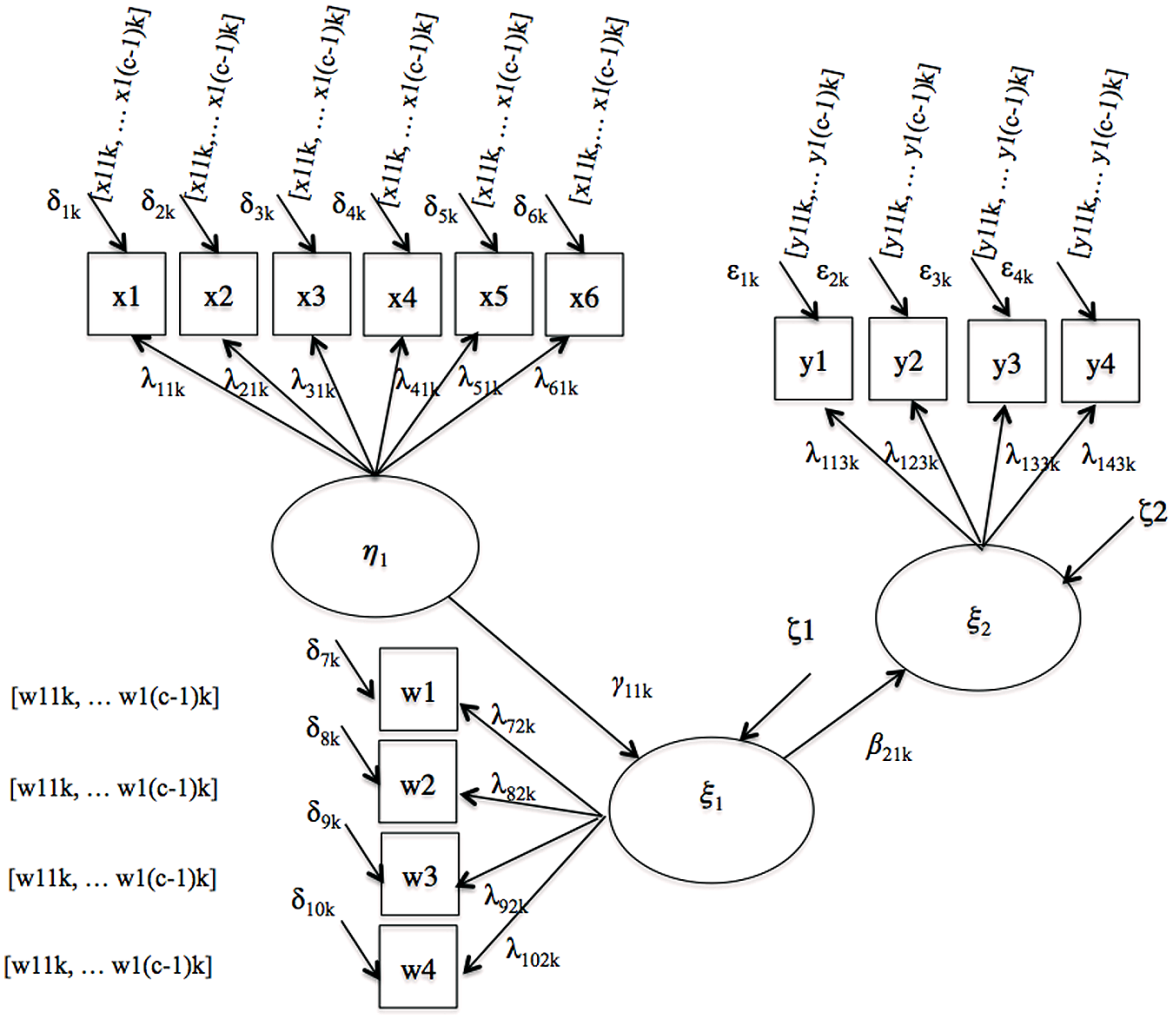
**Mediated regression**.

The third model examined was the mediated regression model illustrated in **Figure 3**. Four regression coefficients capture the structural relationships between variables. One coefficient per group, γ_11,1_ and γ_11,2_, link the predictor variables to the mediators in the referent and the focal group, respectively, and one coefficient per group, β_21,1_ and β_21,2_, link the mediators to the criterion variables.

**FIGURE 4:**
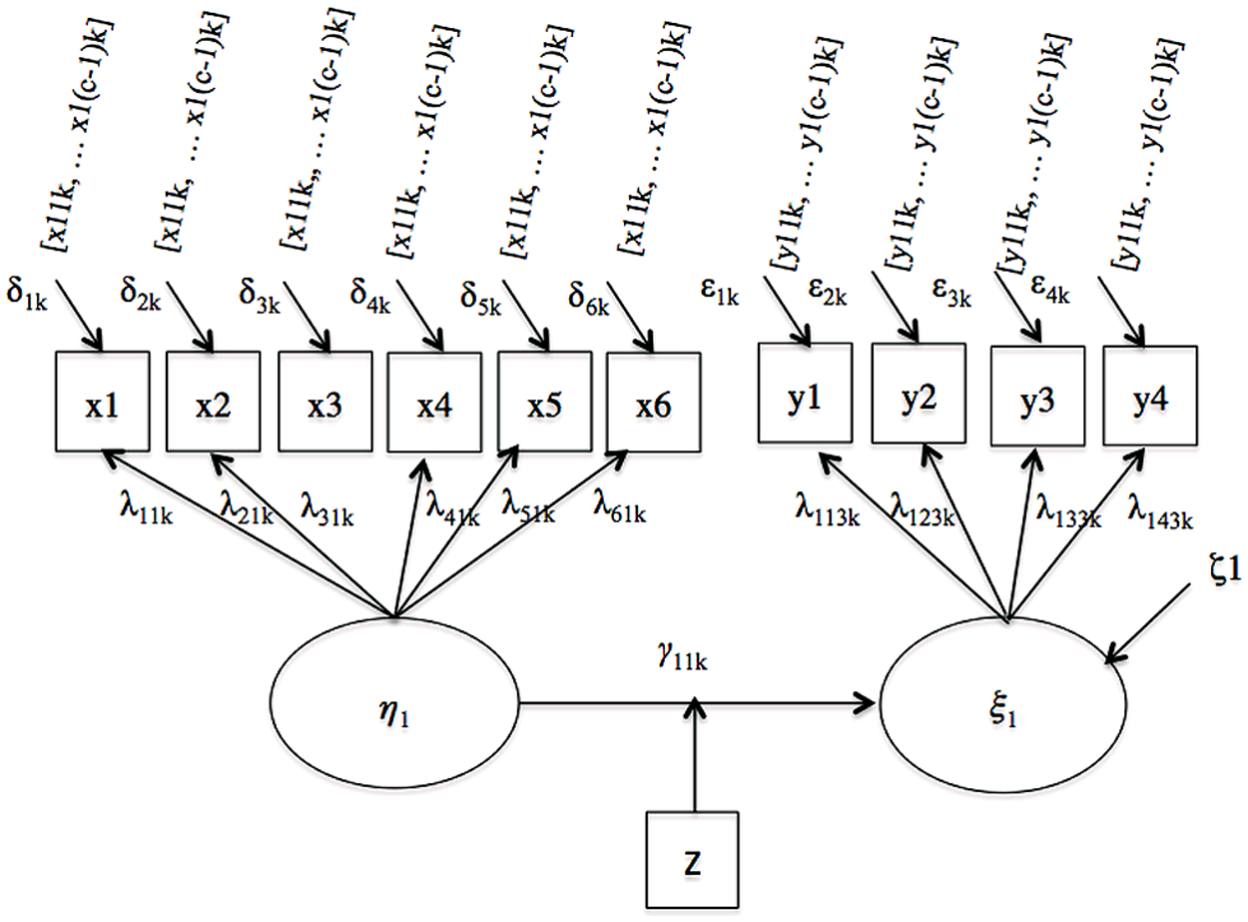
**Moderated regression**.

Finally, we examined a moderated regression model illustrated in **Figure 4**. Two regression coefficients, γ_11,1_ and γ_11,2,_ quantifying the path linking the predictor and criterion latent variables in the referent and the focal group, respectively, summarize the variable relations here.

#### Test length and rating scale

We opted for a six-item measurement model for the target construct in our study. This is consistent with the test length reported by Meade and Lautenschlager (2004a,b) and Kim and Yoon (2011). We chose the polytomous items with three rating categories. This format is common in questionnaire research; for example, three response options (not true – somewhat true – certainly true) are used in the Strengths and Difficulties Questionnaire (Goodman, 1997), among many others. We used four indicators to model the auxiliary latent constructs in structural models, and this decision was not expected to impact the results of the analysis. Four item scales are often used in SEM research because four items is the minimum number of indicators required for a factor to be independently over-identified (Bollen, 1989). For these constructs, we opted for five-point Likert scales. Five point scales are often used due to the increased reliability that more scale points per item affords and is typical in personality questionnaire research (Furnham et al., 2013).

#### Proportion of non-equivalent items

We simulated four levels of invariance: zero non-invariant items (0%), one non-invariant item (16.67%), two non-invariant items (33.33%), and three non-invariant items (50%) out of six for measuring our target construct. With any greater non-invariance than this, researchers would likely be uncomfortable using the scale across subpopulations.

#### Sample size

We fixed sample size in all conditions at 1,000 respondents per group. The effect of sample size is out of scope for the present research, which focuses on model parameters and assumes that there is enough power in the study to estimate them. Limited information estimators that are required for speed in the context of models with categorical indicators are generally acknowledged to require larger sample sizes than linear factor models (Flora and Curran, 2004). Moreover, sample sizes of this magnitude are becoming more and more common in survey research due to advancing data collection technology.

#### Number of replications

A review of previous Monte Carlo research into measurement equivalence revealed that the number of replications ranged between a low of 50 replications per cell by Stark et al. (2006) and high of 500 replications per cell by Kim and Yoon (2011). We executed 1000 replications per cell, the highest number of replications of any of the studies reviewed.

#### Summary of experimental design

The Monte Carlo design involved 2 (modeling approaches)^∗^3 (types of non-invariance)^∗^4 (levels of non-invariance) ^∗^9 (types of structural models = 3 models where the latent variable assumed two structural positions, plus one model where the target latent variable assumed three structural positions) equals 216 conditions.

### CREATING REPRESENTATIVE MODELS

#### Structural coefficient population values

We based the structural components of our models on an empirical study drawn from the applied literature. We searched for an example that contained the four different types of effects typically studied in psychological research, namely, simple regression coefficients, partial regression coefficients, mediated regression coefficients, and moderated regression coefficients. Wasti et al. (2000) presented a study with all four types of coefficient. These authors examined the structural equivalence of the relationship between the antecedents and consequences of sexual harassment between the United States and Turkey.

**FIGURE 5:**
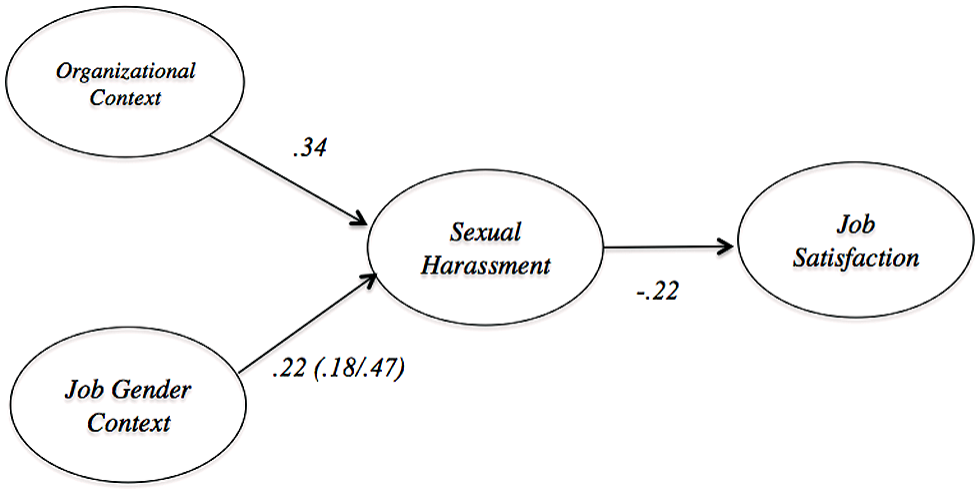
**Nomology of sexual harassment based on Wasti et al. (2000)**.

Sexual harassment was defined as an organizational cause of stress with performance related consequences. It was comprised of gender harassment (e.g., offensive, misogynist remarks), unwanted sexual attention, and sexual coercion. The relevant subsection of the model from Wasti et al. (2000) is shown in **Figure 5**. The model suggests that the two most important determinants of sexual harassment are job-gender context and organizational context. Job-gender context refers to how gender stereotyped the work is, while organizational context describes the features of the organization that communicate acceptance of sexual harassment. Sexual harassment, in turn, is negatively associated with job satisfaction.

We set the population structural regression values for all conditions based on paths from Wasti et al. (2000). The population simple regression effect was set at 0.22, the magnitude of the path between job-gender context and sexual harassment. The population partial regression coefficients were set at 0.22 and 0.37, which are the values of the paths between job-gender context and sexual harassment and organizational climate, respectively. The population mediation structural regression paths were set at 0.22 and -0.22, which correspond to the path from job-gender context to sexual harassment and from sexual harassment and job satisfaction. Wasti et al. (2000) also included the separate estimates of the relations between job-gender context and sexual harassment, at 0.47 for the United States sample and 0.18 for the Turkish sample. These values were used for moderated population structural regression values. The population variances for latent predictor variables were simulated equal to one, while population residual variances of latent criterion variables were simulated equal one minus the square of the structural coefficient.

#### Population factor loadings and simulated non-invariance

All simulated item loadings are presented in Appendix A. Target construct loadings were selected as representative of many questionnaire items using rating scales. These parameters are in the metric used with normal ogive IRT models, where the latent trait is scaled as having the mean of 0 and the variance of 1(i.e., the “theta” parameterization in Mplus; Muthén, 2013). Non-invariance was introduced by reducing focal group loadings on the second, fourth and sixth items by 50%. Our rationale for the 50% effect was that to be detectable in the structural relationship the impact of non-invariance needed to be at least moderate to strong, because existing empirical research suggests the effect of ignoring non-invariance on beta is small (Chen, 2008; Schmitt et al., 2011). For invariant indicators on auxiliary constructs, we followed Meade and Lautenschlager (2004a) by simulating loadings from a normal distribution with mean of 0.6 and a variance of 0.1. Error variances were set at one minus the square of the factor loadings. We then transformed these parameters to the IRT metric for the theta parameterization, by using formulas described by Wirth and Edwards (2007). While several authors have made a distinction between mixed and invariant patterns of loading differences across groups, Chen’s (2008) literature review found just 7% of studies revealed mixed patterns of non-invariance. We focused on the so-called uniform pattern of invariance, the dominant outcome whereby all non-invariant items are lower in the focal group.

#### Population thresholds and simulated non-invariance

All simulated item thresholds are presented in Appendix A. Item thresholds of the target construct were representative of many questionnaire items using rating scales. We simulated non-equivalence on the second, fourth and sixth indicators on the focal construct by subtracting 0.8, 1.00, and 1.2 from the bottom threshold of the referent group. This did not disturb the relative ordering of the thresholds. For the auxiliary constructs, we followed Meade and Lautenschlager (2004b) to create thresholds by first drawing the lowest threshold from a normal distribution with mean of -1.7 and a standard deviation of 0.45. The remaining three thresholds were then created by adding constants to the lowest threshold for each item to give four thresholds per item. The constants were 1.2, 2.4, and 3.6. Threshold and loadings were transformed to the IRT metric using formulas from Wirth and Edwards (2007).

## MATERIALS AND METHOD

### ANALYSES

#### Model identification

To identify the metric of the latent factors, the loading of the first item of each factor was fixed at its population value in both groups. This allowed the latent variable variances and residual variances to be freely estimated in both groups. The means / intercepts of the latent factors were set to zero in the referent group and estimated freely in the focal group.

#### Specification of invariant and non-invariant conditions

Under the theta parameterization in Mplus the model where measurement invariance is imposed sees thresholds and loadings constrained equal across groups, error variances fixed at one in the referent group and free in the focal group, and factor means fixed at zero in the referent group and freely estimated in the focal group. This setup was adopted in conditions where measurement non-invariance was ignored. The alternative models had the loadings and / or thresholds for the items known to be non-invariant freely estimated across groups.

#### Estimation

All models were fitted to polychoric correlations of the simulated item responses in MPlus 7.11 (Muthén and Muthén, Muthén and Muthén, 1998–2010) using the diagonally weighted least squares (DWLS) estimator with robust standard errors (denoted WLSMV in MPlus). Several simulation studies have shown that this estimator compares favorably to full information maximum likelihood (FIML) in comparable contexts to the current study (Muthén et al., Unpublished; Flora and Curran, 2004; Beauducel and Herzberg, 2006; Forero and Maydeu-Olivares, 2009). The simulations were executed by calling MPlus from the statistical computing environment R 3.0 using the package MPlusAutomation (Hallquist, 2011). All MPlus and R scripts are available at the following link: http://figshare.com/articles/Apples_Oranges_Monte_Carlo_Study/1060341.

#### Model performance

We examined five indicators of model performance. These included (1) the proportion of non-converged and inadmissible solutions; (2) how well the empirical chi-square distribution approximated the theoretical chi-square distribution; (3) the impact of the experimental conditions on power to reject the null hypothesis that the regression parameters were not significantly different from zero; (4) the relative bias, defined as the observed regression parameter minus the true parameter divided by the true parameter (Schunn and Wallach, 2005), and finally; (5) the coverage rate for all regression coefficients. Following Forero and Maydeu-Olivares (2009), we interpreted relative bias of less than 10% as acceptable, between 10 and 20% as substantial, and greater than 20% as unacceptable, and we considered coverage acceptable where the true parameter was captured by between 92.5 and 97.5% of 95% confidence intervals. We describe our results in the text; where boundary points are visible, we present results graphically.

## RESULTS

### MODEL ADMISSIBILITY AND GLOBAL FIT

Broad patterns observed following execution of the simulations greatly simplify presentation of results. First, all models converged to admissible solutions, indicating that the simulations ran well. We do not discuss model convergence further. The large sample size meant that power to detect whether regression parameters were significantly different from zero remained above 90% for all conditions of the study. We do not discuss power further. A clear difference emerged between the global fit results for models where increasing levels of non-invariance was modeled and where it was ignored. In all conditions where the non-invariance was modeled, the χ^2^ test consistently approximated the theoretical chi-square distribution well at the first, second, fifth, and tenth percentiles. When the increasing level of non-invariance was ignored, χ^2^ correctly rejected all models. We do not discuss χ^2^ goodness of fit further.

We turn now to discuss relative bias and coverage of regression coefficients for all conditions. These results summarized in **Tables 1** and **Table 2**. They are also graphically summarized and are available at http://dx.doi.org/10.6084/m9.figshare.1060341. However, we include a few typical graphical illustrations in the results sections that follow.

**Table 1:** Relative bias for ignored non-invariance conditions.

	Thresholds				Slopes				Thresholds and Slopes			
Items	γ_11,1_		γ_11,2_		γ_11,1_		γ_11,2_		γ_11,1_		γ_11,2_	
**Simple,predictor**
0	0.00		–0.01		0.00		0.01		0.00		0.01	
1	–0.03		0.04		–0.05		0.06		–0.07		0.13	
2	–0.04		0.09		–0.09		0.16		–0.12		0.35	
3	–0.03		0.11		–0.11		0.23		–0.11		0.44	
**Simple,criterion**
0	0.00		0.01		0.01		0.01		0.01		0.00	
1	0.03		–0.03		0.06		–0.06		0.09		–0.11	
2	0.06		–0.06		0.13		–0.13		0.15		–0.25	
3	0.06		–0.07		0.14		–0.18		0.13	–0.30												

**Items**	**γ_11,1_**	**γ_12,1_**	**γ_11,2_**	**γ_12,2_**	**γ_11,1_**	**γ_12,1_**	**γ_11,2_**	**γ_12,2_**	**γ_11,1_**	**γ_12,1_**	**γ_11,2_**	**γ_12,2_**

**Partial,predictor**
0	0.01	0.01	0.01	0.01	0.01	0.00	0.02	0.01	0.01	0.01	0.00	0.01
1	–0.03	0.00	0.05	0.01	–0.05	0.01	0.07	0.01	–0.08	0.00	0.13	0.01
2	–0.05	0.00	0.09	0.01	–0.09	0.01	0.17	0.01	–0.12	0.01	0.34	0.01
3	–0.04	0.01	0.10	0.00	–0.11	0.00	0.23	0.00	–0.13	0.00	0.44	0.01
**Partial,criterion**
0	0.01	0.01	0.00	0.02	0.00	0.01	0.00	0.01	0.01	0.01	0.01	0.01
1	0.04	0.05	–0.03	–0.03	0.07	0.06	–0.05	–0.05	0.09	0.10	–0.10	–0.10
2	0.06	0.07	–0.05	–0.07	0.12	0.12	–0.12	–0.14	0.16	0.15	–0.24	–0.25
3	0.05	0.04	–0.06	–0.06	0.14	0.13	–0.19	–0.19	0.14	0.15	–0.29	–0.29

**Items**	**γ_11,1_**	**β_21,1_**	**γ_11,2_**	**β_21,2_**	**γ_11,1_**	**β_21,1_**	**γ_11,2_**	**β_21,2_**	**γ_11,1_**	**β_21,1_**	**γ_11,2_**	**β_21,2_**

**Mediation,predictor**
0	0.00	0.01	0.00	0.01	0.01	0.01	0.01	0.00	0.02	0.02	0.02	0.01
1	–0.03	0.01	0.04	0.00	–0.05	0.01	0.07	0.01	–0.08	0.00	0.12	0.02
2	–0.04	0.01	0.09	0.01	–0.09	–0.09	0.18	0.01	–0.13	0.02	0.32	0.00
3	–0.03	0.01	0.09	0.00	–0.11	0.01	0.22	0.01	–0.11	0.03	0.43	0.01
**Mediation,mediator**
0	0.00	0.02	0.00	0.00	0.01	0.01	0.00	0.00	0.00	0.02	0.00	0.01
1	0.04	–0.02	–0.03	0.04	0.05	–0.04	–0.06	0.05	0.10	–0.09	–0.09	0.12
2	0.05	–0.04	–0.07	0.09	0.10	–0.10	–0.13	0.17	0.16	–0.12	–0.26	0.33
3	0.06	–0.04	–0.07	0.10	0.14	–0.12	–0.19	0.23	0.13	–0.12	–0.31	0.45
**Mediation,criterion**
0	0.01	0.02	0.00	0.01	0.01	0.02	0.00	0.01	0.02	0.01	0.00	0.01
1	0.01	0.05	–0.01	–0.02	0.01	0.07	0.01	–0.06	0.01	0.09	0.02	–0.10
2	0.01	0.07	0.00	–0.07	0.01	0.10	0.01	–0.14	0.00	0.15	0.00	–0.25
3	0.01	0.06	–0.01	–0.06	0.01	0.14	0.01	–0.18	0.02	0.14	0.01	–0.31
**Moderation,predictor**
0	0.01		0.01		0.01		0.00		0.02		0.00	
1	–0.03		0.04		–0.04		0.06		–0.07		0.11	
2	–0.04		0.08		–0.10		0.15		–0.14		0.30	
3	–0.04		0.09		–0.13		0.20		–0.14		0.39	
**Moderation,criterion**
0	0.00		0.02		0.01		0.00		0.02		0.01	
1	0.04		–0.03		0.07		–0.05		0.11		–0.10	
2	0.08		–0.06		0.12		–0.13		0.17		–0.23	
3	0.05		–0.07		0.16		–0.17		0.19		–0.27	

*Regression parameter labels correspond to **Figures 1–4**; group 1 is the referent group, group 2 is the focal group.*

**Table 2:** Coverage rates for ignored non-invariance.

	Thresholds				Slopes				Thresholds and Slopes			
Items	γ_11,1_		γ_11,2_		γ_11,1_		γ_11,2_		γ_11,1_		γ_11,2_	
**Simple,predictor**
0	0.95		0.96		0.95		0.95		0.95		0.95	
1	0.94		0.95		0.93		0.94		0.92		0.93	
2	0.93		0.96		0.90		0.94		0.86		0.82	
3	0.92		0.94		0.89		0.90		0.88		0.75	
** Simple,criterion**
0	0.95		0.93		0.95		0.96		0.96		0.96	
1	0.95		0.93		0.94		0.92		0.94		0.89	
2	0.95		0.92		0.94		0.83		0.92		0.62	
3	0.95		0.92		0.94		0.77		0.95		0.50	

**Items**	**γ_11,1_**	**γ_12,1_**	**γ_11,2_**	**γ_12,2_**	**γ_11,1_**	**γ_12,1_**	**γ_11,2_**	**γ_12,2_**	**γ_11,1_**	**γ_12,1_**	**γ_11,2_**	**γ_12,2_**

**Partial,predictor**
0	0.95	0.93	0.95	0.95	0.93	0.93	0.96	0.95	0.95	0.95	0.94	0.95
1	0.94	0.95	0.94	0.94	0.93	0.94	0.95	0.95	0.89	0.95	0.93	0.95
2	0.93	0.95	0.95	0.95	0.88	0.95	0.91	0.94	0.86	0.95	0.82	0.95
3	0.94	0.95	0.95	0.95	0.87	0.95	0.89	0.93	0.86	0.95	0.75	0.94
**Partial,criterion**
0	0.95	0.95	0.94	0.95	0.95	0.93	0.94	0.95	0.95	0.94	0.95	0.95
1	0.94	0.95	0.94	0.93	0.95	0.93	0.94	0.95	0.95	0.94	0.89	0.84
2	0.96	0.95	0.92	0.88	0.95	0.93	0.94	0.95	0.91	0.91	0.65	0.42
3	0.94	0.94	0.93	0.89	0.95	0.93	0.94	0.95	0.93	0.92	0.50	0.28
**Mediation,predictor**
0	0.95	0.96	0.95	0.95	0.95	0.94	0.95	0.94	0.95	0.95	0.95	0.96
1	0.94	0.95	0.96	0.94	0.93	0.95	0.95	0.95	0.91	0.95	0.95	0.95
2	0.94	0.95	0.94	0.95	0.91	0.91	0.91	0.95	0.87	0.94	0.84	0.94
3	0.92	0.94	0.94	0.95	0.89	0.95	0.90	0.96	0.87	0.94	0.78	0.95
**Mediation, mediator**
0	0.94	0.96	0.94	0.94	0.95	0.95	0.94	0.95	0.95	0.95	0.96	0.95
1	0.95	0.94	0.94	0.95	0.94	0.92	0.92	0.95	0.96	0.89	0.88	0.93
2	0.95	0.95	0.92	0.94	0.94	0.91	0.87	0.91	0.92	0.85	0.61	0.84
3	0.94	0.95	0.93	0.94	0.93	0.88	0.76	0.88	0.94	0.87	0.49	0.74
**Mediation,criterion**
0	0.95	0.95	0.95	0.95	0.94	0.95	0.94	0.94	0.94	0.92	0.94	0.95
1	0.95	0.94	0.94	0.94	0.93	0.95	0.94	0.91	0.95	0.95	0.95	0.88
2	0.94	0.94	0.94	0.94	0.95	0.95	0.95	0.85	0.96	0.92	0.95	0.63
3	0.95	0.94	0.93	0.93	0.94	0.95	0.95	0.77	0.95	0.92	0.95	0.48

**Items**	**γ_11,1_**	****	**γ_11,2_**	****	**γ_11,1_**	****	**γ_11,2_**	****	**γ_11,1_**	****	**γ_11,2_**

**Moderation,predictor**
0	0.95		0.96		0.94		0.93		0.95		0.94	
1	0.94		0.94		0.94		0.95		0.92		0.92	
2	0.94		0.93		0.91		0.88		0.87		0.62	
3	0.94		0.93		0.87		0.79		0.87		0.44	
**Moderation,criterion**
0	0.95		0.95		0.94		0.95		0.94		0.95	
1	0.96		0.92		0.94		0.88		0.94		0.79	
2	0.96		0.89		0.94		0.74		0.94		0.38	
3	0.96		0.83		0.93		0.55		0.93		0.18	

### MODELED NON-INVARIANCE ON THRESHOLD, LOADING, AND COMBINED CONDITIONS

In the conditions where the measurement non-invariance was modeled, the regression parameters had acceptable coverage and relative bias. Based on this pattern, we further simplify reporting of results, describing coverage and relative bias only for models where the measurement non-invariance was ignored.

### IGNORED NON-INVARIANCE OF THRESHOLDS

**FIGURE 6:**
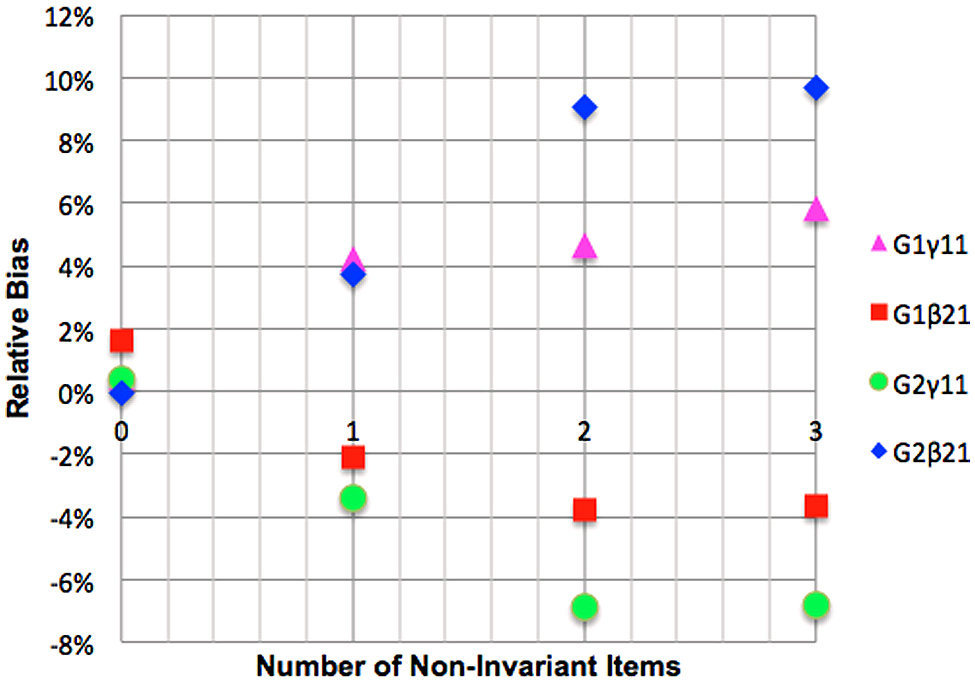
**Impact of mediator variable threshold non-invariance on relative bias of mediation betas**.

Under the threshold only non-invariance condition when non-invariance was ignored, relative bias always fell into the range defined as acceptable, i.e., less than 10% (one cell of the design showed 11%). When the non-invariance existed in the latent predictor, positive relative bias was observed in the focal group indicating over-estimation of the regression coefficient. When the latent variable with non-invariance occupied the mediator position, the path linking the predictor to the mediator in the focal group was overestimated and the path linking the mediator to the ultimate criterion was underestimated. The opposite patterns of bias to those just described were observed in the referent group. The result for this condition is illustrated in **Figure 6**.

The effects of ignoring threshold only non-invariance on coverage, for the most part, parallel the results for relative bias. That is, there were minimal negative effects on parameter recovery for the regression coefficient. The coverage was acceptable in all but a small handful of conditions, i.e., with between 92.5 and 97.5% of 95% confidence intervals containing the true parameter. There was no discernible pattern in relation to whether the non-invariant construct occupied the position of the predictor or the criterion, or to whether the departure from acceptable coverage was on the target construct that exhibited bias or, in the case of the partial and mediated models, involved measurement invariant constructs. We thus conclude that structural coefficient coverage should not be a primary concern for researchers ignoring non-invariant item thresholds.

### IGNORED NON-INVARIANCE OF LOADINGS

#### Simple regression with predictor non-invariance

Ignoring loading only non-invariant items led to acceptable negative relative bias for γ_11,1_ (referent group) when one or two non-invariant loading were ignored, and substantial negative relative bias when three non-invariant loadings were ignored. Ignoring a single non-invariant item led to positive but acceptable relative bias for the regression parameter γ_11,2_ (focal group). Bias for the focal group became positive and substantial for two non-invariant loadings, and unacceptable for three non-invariant loadings. Coverage for γ_11,1_ fell slightly below acceptable levels when two and three non-invariant items are ignored, and for γ_11,2_, it fell slightly below acceptable when three non-invariant loadings were ignored_._

#### Simple regression with criterion non-invariance

Relative bias for γ_11,1,_ (referent group) was positive but acceptable for one ignored non-invariant loading and substantial and positive for two and three ignored non-invariant loadings. Non-invariant items led to negative relative bias for γ_11,2_ (focal group) that was acceptable for a single item and substantial for two and three non-invariant items. Coverage for γ_11,1_ was acceptable for all ignored non-invariance. Coverage for γ_11,2_ dropped to an unacceptable level for even a single non-invariant item and progressively worsened with further ignored non- invariance.

#### Partial regression with predictor non-invariance

The referent group parameter γ_11,1_ was characterized by negative but acceptable relative bias for one or two ignored non-invariant loadings, and substantial relative bias for three non-invariant items. The focal group parameter γ_11,2_ showed acceptable positive relative bias for a single non-invariant item, reaching substantial and unacceptable levels of positive bias for two and three non-invariant items. Relative bias for γ_12,1_ and γ_12,2_ was acceptable across all levels of ignored non-invariance. Coverage for γ_12,1_ and γ_12,1_ remained acceptable for all levels of ignored non-invariance while coverage for both γ_12,2_ and γ_12,2_ fell just below acceptable levels when two or three non-invariant items were ignored.

#### Partial regression with criterion non-invariance

Relative bias for the referent group parameter γ_11,1_ was positive and acceptable for one ignored non-invariant loading, and substantial for two and three ignored non-invariant loadings. The focal parameter γ_11,2_ showed acceptable negative relative bias with one ignored non-invariant item, and substantial negative relative bias for two ignored non-invariant items, and unacceptable relative bias for three ignored non-invariant items. Relative bias for γ_12,1_ was positive but acceptable for one ignored non-invariant item, and substantial for two or three ignored non-invariant items. Relative bias for γ_12,2_ was negative and acceptable for one invariant item and substantial for two and three non-invariant items. Coverage for γ_11,1_ and γ_12,1_ was acceptable for all levels of ignored non-invariance. The same was true for γ_11,2_ and γ_12,2_.

#### Mediated regression with predictor non-invariance

Relative bias for the referent group parameter γ_11,1_ was negative but acceptable for one and two ignored non-invariant loadings and substantial for three non-invariant loadings. Relative bias for the focal group parameter γ_11,2_ was acceptable for a single non-invariant item, substantial for two non-invariant items and unacceptable for three non-invariant items. Relative bias of the coefficients β_21,1_ and β_21,2_ was acceptable for all levels of non-invariance. Coverage for all regression coefficients in referent and focal groups was acceptable except for γ_11,1_ and γ_11,2_ when two or three invariant items were ignored and β_21,1_ and β_21,2_ when two and three non-invariant items were ignored.

#### Mediated regression with mediator non-invariance

**FIGURE 7:**
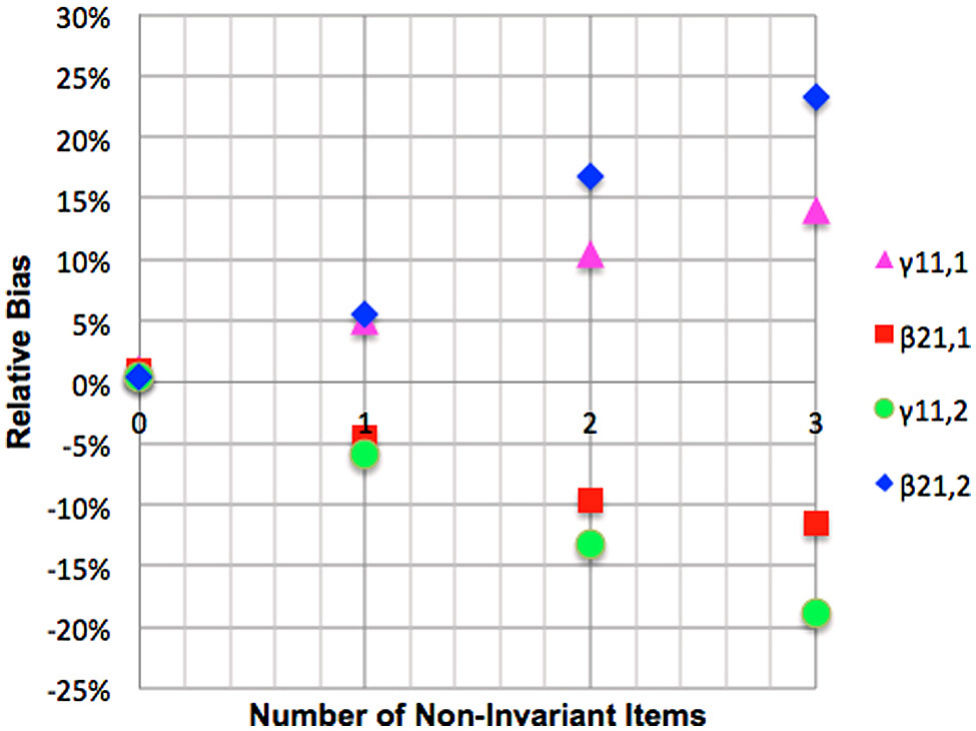
**Impact of mediator variable loading non-invariance on relative bias of mediation betas**.

Relative bias for the referent group parameter γ_11,1_ was positive but acceptable for one ignored non-invariant loading and substantial for two and three ignored non-invariant loadings. Relative bias for the focal group parameter γ_11,2_ was negative and acceptable for a single non-invariant loading and substantial for two and three ignored non-invariant loadings. Relative bias of β_21,1_ was negative and acceptable for one and substantial for two and three ignored non-invariant loadings, while relative bias for β_21,2_ was positive and acceptable for a single non-invariant loading, substantial for two and unacceptable for three ignored non-invariant items. These results are presented graphically in **Figure 7**. Coverage rates for γ_11,1_ were acceptable. Coverage for β_21,1_ was acceptable for a single ignored loading but unacceptable for two and three ignored loadings. Coverage for γ_11,2_ and β_21,2_ reached unacceptable levels when two or three non-invariant items were ignored.

#### Mediated regression with criterion non-invariance

Relative bias for the referent group parameter γ_11,1_ was positive and acceptable. Relative bias for the focal group parameter γ_11,2_ was also positive and acceptable. Relative bias for β_21,1_ was positive and acceptable for one ignored non-invariant loading, and substantial for two or three ignored non-invariant loadings. Relative bias for β_21,2_ was negative and acceptable for one non-invariant item, becoming substantial for two and three non-invariant items. Coverage rates for all coefficients were acceptable except for β_21,2_ where it became unacceptable when a single non-invariant items was ignored.

#### Moderated regression with predictor non-invariance

Relative bias for the referent group parameter γ_11,1_ was positive but acceptable for one ignored non-invariant loadings and substantial for two and three ignored non-invariant items. Relative bias for the focal group parameter γ_11,2_ was negative and acceptable for one ignored non-invariant item and substantial for two and three ignored non-invariant items. Coverage for γ_11,1_ was acceptable for one and two ignored non-invariant items but unacceptable for three ignored non-invariant loadings, and the same was observed for γ_11,2_.

#### Moderated regression with criterion non-invariance

Bias for the referent group parameter γ_11,1_ was positive and acceptable for one ignored non-invariant loading and substantial for two and three items. Relative bias for the focal group parameter γ_11,2_ was negative. It was acceptable for one and two ignored non-invariant items and substantial for three items. Coverage for γ_11,1_ became unacceptable when two non-invariant items were ignored. Similarly, the coverage for γ_11,2_ fell to unacceptable levels when two or three non-invariant items were ignored.

### THRESHOLD AND LOADING NON-INVARIANCE

#### Simple regression with predictor non-invariance

Non-invariant items caused negative relative bias for the referent group parameter γ_11,1_. This bias was acceptable for one ignored non-invariant item and substantial for two and three ignored non-invariant items. Ignoring a single non-invariant item led to positive and substantial relative bias for the focal group regression parameter γ_11,2_. This positive bias was unacceptable when two or three non-invariant items were ignored. Coverage for γ_11,1_ was unacceptable for all levels of ignored non-invariance. Coverage for γ_11,2_ deteriorated to unacceptable levels with two non-invariant items.

#### Simple regression with criterion non-invariance

Relative bias for γ_11,1_ was positive and ranged from acceptable for a single non-invariant item to substantial for two and three non-invariant items. Non-invariant items led to negative relative bias for γ_11,2_ that was substantial for a single item and unacceptable for two and three ignored non-invariant items. Coverage for the regression parameter γ_11,1_ was acceptable for all ignored non-invariance except for two ignored items when it was marginally unacceptable. Coverage for γ_11,2_, however, dropped to unacceptable levels as soon as a single non-invariant item is ignored. Coverage progressively worsened with further ignored non-invariance.

#### Partial regression with predictor non-invariance

Relative bias for γ_11,1_ was negative and acceptable for a single item and substantial for two and three items. γ_11,2_ suffered from substantial positive relative bias when even a single ignored non-invariant item was ignored. Relative bias increased to unacceptable levels for two and three non-invariant items. The relative bias of coefficients γ_12,1_ and γ_12,2_ was acceptable. Coverage for γ_12,1_ and γ_12,2_ remained acceptable for all levels of ignored non-invariance. However, the coverage rate for γ_11,1_ was unacceptable for even one ignored non-invariant item, and coverage for γ_11,2_ became unacceptable when two or three non-invariant items were ignored.

#### Partial regression with criterion non-invariance

Relative bias for γ_11,1_ was positive and acceptable for one ignored item, becoming substantial when two or three items were ignored. γ_12,1_ suffered from substantial negative relative bias with when one, two or three non-invariant items are ignored. Relative bias for γ_11,2_ was negative and substantial for a single ignored item, and negative and unacceptable when two or three items were ignored. Relative bias for γ_12,2_ was substantial when a single non-invariant item was ignored and unacceptable when two or three such items were ignored. Coverage for referent group parameters γ_11,1_ and γ_12,1_ became unacceptable when even a single non-invariant item was ignored and the coverage for γ_11,2_ and γ_12,2_ fell to unacceptable levels as soon as a two or three non-invariant items were ignored.

#### Mediated regression with predictor non-invariance

Relative bias for γ_11,1_ was negative and acceptable for a single ignored non-invariant item and substantial for two or three ignored non-invariant items. Relative bias for this coefficient in the focal group, γ_11,2_ was positive and substantial for a single ignored item and unacceptable when two or three non-invariant items are ignored. Relative bias of the coefficients β_21,1_ and β_21,2_ was near zero for all levels of non-invariance. Coverage for γ_11,1_ fell below acceptable when one, two and three items are ignored, while the rate for γ_11,2_ falls also falls below the acceptable threshold when two or three non-invariant items are ignored. Coverage for coefficients β_21,1_ and β_21,2_ remained acceptable for all levels of invariance.

#### Mediated regression with mediating non-invariance

**FIGURE 8:**
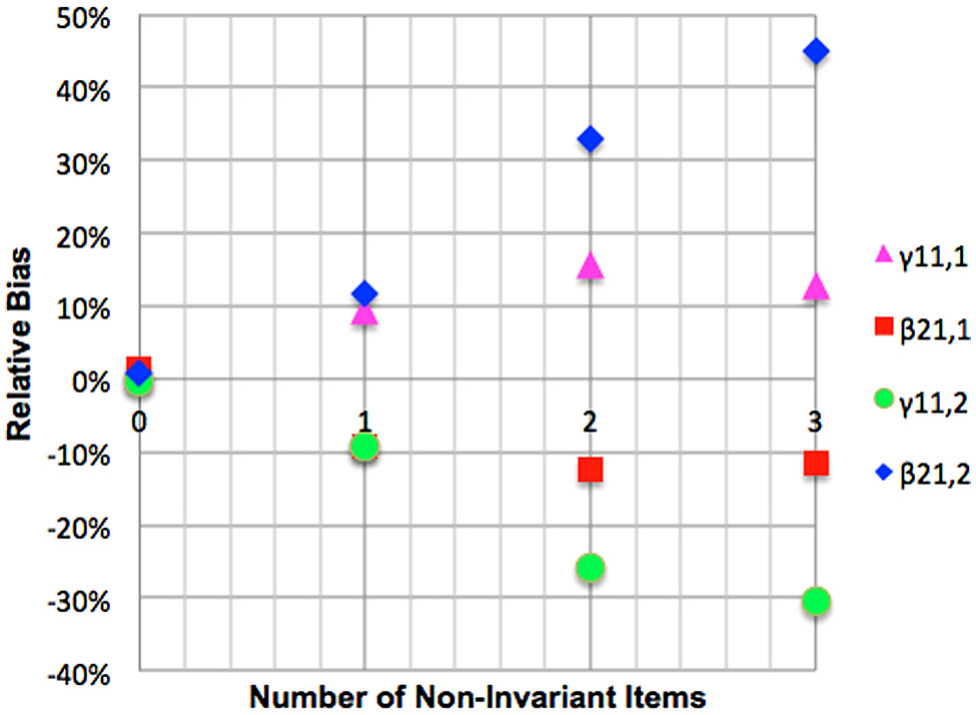
**Impact of mediator variable combined non-invariance on relative bias of mediation betas**.

Relative bias for γ_11,1_ was positive and substantial for one, two or and three invariant items. Relative bias for γ_11,2_ was negative and acceptable with one non-invariant item, worsening to unacceptable further non-invariance. Relative bias of β_21,1_ was negative and acceptable for a single item and substantial for two or three items. Relative bias for β_21,2_ was positive and substantial for one non-invariant item and this worsened with further non-invariance to unacceptable levels for two and three items. These results are presented graphically in **Figure 8**. Coverage rates for γ_11,1_ with one ignored non-invariant item and unacceptable for two or three items. Coverage rates for γ_11,2_ are unacceptable when even a single non-invariant item is ignored. Coverage rates for β_21,1_ are also unacceptable when one or more non-invariant items are ignored while coverage for β_21,2_ is unacceptable when two or three non-invariant item are ignored.

#### Mediated regression with ultimate criterion non-invariance

Relative bias for γ_11,1_ was acceptable. Relative bias for γ_11,2_ was also acceptable. Relative bias for β_21,1_ was positive and acceptable when one non-invariant item was ignored but positive and substantial when two or three non-invariant items are ignored. Relative bias for β_21,2_ was negative and substantial with one non-invariant item and unacceptable for two or three non-invariant items. The coverage rate for all regression coefficients in this condition is acceptable except for β_21,2_ where it deteriorates to unacceptable levels as soon as two non-invariant items are ignored and β_21,1_ where coverage is unacceptable for one, two and three ignored non-invariant items.

#### Moderated regression with predictor non-invariance

When the non-invariant construct is the predictor, relative bias for γ_11,1_ is negative but acceptable for a single item and substantial for two and three items. Relative bias for γ_11,2_ is positive and substantial for a single ignored non-invariant item and becomes unacceptable for two and three ignored non-invariant items. Coverage for γ_11,1_ and γ_11,2_ became unacceptable as soon as a single non-invariant item was ignored and progressively worsened with increased ignored non-invariance.

#### Moderated regression with criterion non-invariance

Relative bias for γ_11,1_ was positive and substantial for one to three ignored non-invariant items. Relative bias for γ_11,2_ was negative and substantial as soon as a single non-invariant item is ignored becoming unacceptable when two or three items are ignored. Coverage for γ_11,1_ is acceptable, while in the focal group the coverage for γ_11,2_ falls away to unacceptable levels as soon as any non-invariance is ignored, worsening with further ignored non-invariance.

## DISCUSSION

The issue of measurement invariance is important to any research or practice setting where the same measurement instrument is being used to assess individuals from different populations. Until now the focus of methodological work, looking at strategies for dealing with non-invariant measurement has mainly been restricted to measurement models and latent mean differences, with notable exceptions from Chen (2008) and Oberski (2014). The focus of the current article was on the implications of ignoring measurement non-invariance for accurate recovery of regression coefficients in full structural equation models. The results for the threshold conditions, loading conditions, and threshold and loading conditions showed that unacceptable relative bias and coverage were limited to the focal group regression parameter. While bias was observed for referent group parameters, this was never unacceptable. This pattern holds across simple, partial, mediated and moderated regression models. Under the conditions studied, i.e., lower focal group loadings, an acquiescent response style, or both, any path going into the non-invariant factor will yield an overestimated regression coefficient in the focal group, while any path coming out of the non-invariant factor will yield an underestimated regression coefficient in the focal group. The bias in the regression parameters emerged due to errors in the estimation of latent variances due to ignoring non-invariance. When predictor non-invariance is ignored due to lower focal loadings or acquiescent responding, focal regression coefficients are over-estimated (i.e., relative bias is positive) and when criterion non-invariance is ignored focal regression coefficients are under-estimated (i.e., relative bias is negative). When the non-invariant latent construct is in the mediator position, we see the path to it under-estimated and from it to the ultimate criterion variable overestimated. The aforementioned patterns were reversed in the referent group

### IMPLICATIONS OF RESULTS REGARDING THRESHOLD NON-INVARIANCE

When either one or two items with non-invariant thresholds were not modeled, relative bias occurred in the aforementioned directions. However, ignoring the non-invariance led to relative bias below 10%, a level considered acceptable by Forero and Maydeu-Olivares (2009). Coverage rates were relatively unaffected. It is tempting for the applied researcher to conclude that they can ignore threshold non-invariance with impunity and argue that measures are consistent both across groups and past studies unless the number of non-invariant items is extreme. However, researchers must be careful to note that this is only the case if latent means are not a focus of the research (Steinmetz et al., 2009).

### IMPLICATIONS OF RESULTS REGARDING LOADING NON-INVARIANCE

Across all types of structural models ignoring predictor non-invariance leads to over-estimation of focal regression coefficients, ignored criterion non-invariance leads to underestimation of focal regression coefficients, and when the non-invariant latent variable is in the mediating position the path to the mediator is underestimated while the path from the mediator in overestimated. The important difference between the loading only and threshold only conditions is that whereas relative bias never hit unacceptable levels in the threshold only condition, the relative bias in the loading condition routinely exceeded substantial and unacceptable thresholds when three non-invariant loadings were not modeled. Whereas coverage was not an issue for threshold only non-invariance, coverage became an issue in the loading only condition. The implications for the researcher are that ignoring non-invariance to permit scale comparability with previous research is okay for a single item with a non-invariant loading. However, when the non-invariance is on the loadings of two or more items and relational invariance is of critical importance, modeling the non-invariance is the best approach.

### IMPLICATIONS OF RESULTS REGARDING LOADING AND THRESHOLD NON-INVARIANCE

Again we see consistency with the general pattern of the impact of ignored non-invariance on predictor latent variables leading to focal group positive relative bias, non-invariance on criterion latent variables leading to negative relative bias, and non-invariance on mediating latent variables producing mixed relative bias consistent with the role of the target variable (either independent or dependent). The main difference here is that the non-invariance causes problems for relative bias in the discussed directions at even lower levels of ignored non-invariance than for the thresholds only and slope only conditions. Serious problems are observed for relative bias when even one item with non-invariant loading and thresholds is not freely estimated across groups. From this early stage the estimation accuracy of the regression parameters have unacceptable relative bias, a problem that worsens with further ignored non-invariance.

### LIMITATIONS, FUTURE DIRECTIONS, AND CONCLUSION

This study only simulated data for three-point scales. While this number of scale points is regularly used in non-cognitive research, it is important that these results eventually be extended to dichotomous rating scales used in cognitive ability questionnaires, for example. This study also only simulated measurement invariance conditions for scales comprized of six items. While past Monte Carlo studies of measurement equivalence have also used six item scales, it would be beneficial to include the effect of ignoring measurement non-invariance on longer scales in terms of model recovery of regression parameters using alternate methods that are sometimes recommended to deal with longer scales in structural models (e.g., Yang et al., 2009).

We studied the impact of ignoring lower focal group loadings and focal group acquiescence. While Chen (2008) found lower focal group loadings were observed in over 90% of cases of measurement non-invariance, and acquiescence is a common response style, examining other conditions such as mixed loading non-invariance and extreme response styles are also important directions for future research. The current study also examined the impact of ignoring measurement non-invariance on regression parameter recovery assuming the distribution of the underlying latent variables is multivariate normal. It will be interesting to examine whether the results shown here generalize to conditions where this assumption is violated (c.f., DeMars, 2012). Finally, it is also important to examine the accuracy of Oberski’s (2014) method in the context of regressions between factors indicated by categorical items.

Despite these limitations, the current study has important practical implications for researchers measuring constructs across multiple populations. The principal message from this study is that researchers must take the issue of measurement equivalence of the measures of latent variables seriously if they are interested in accurately estimating between construct relations using latent regression models. This is evident from the deteriorating trend in the accuracy of regression parameters as more non-invariance was introduced into the models. The current special issue and a rapidly expanding literature on measurement invariance both suggest that statisticians and psychometrics experts take the issue of measurement invariance extremely seriously. No doubt numerous applied researchers have caught themselves asking the question “does it really matter?” The short answer is to this question is “yes.”

## Conflict of Interest Statement

The authors declare that the research was conducted in the absence of any commercial or financial relationships that could be construed as a potential conflict of interest.

## Footnotes

1 There is a well-developed literature on relational equivalence where regressions are conditioned on the observed variable composite. See, for example Drasgow (1982, 1984) and the duality theorems of Millsap (1995, 1998).

## APPENDIX A. MONTE CARLO MEASUREMENT MODEL PARAMETERS

**Table A1:** Loadings.

	Target construct		Auxiliary construct	
Item	Referent	Focal	Both
1	0.85	0.85	0.55
2	1.40	0.70	1.40
3	1.25	1.25	1.10
4	2.00	1.00	0.80
5	0.50	0.50	–
6	0.75	0.38	–

**Table A2:** Thresholds.

	Target construct				Auxiliary construct			
	Referent		Focal		Both			
Item	*t*1	*t*2	*t*1	*t*2	*t*1	*t*2	*t*3	*t*4
1	–1.70	1.50	–1.70	1.50	–2.00	–0.80	0.20	1.70
2	–0.40	1.90	–1.20	1.90	–2.50	–0.90	0.30	1.90
3	0.70	2.30	0.70	2.30	–1.50	–0.20	0.80	2.30
4	–0.45	2.75	–1.45	2.75	–1.70	0.30	1.00	2.50
5	0.80	2.20	0.80	2.20	–	–	–	–
6	1.20	2.00	–0.20	2.00	–	–	–	–

## References

[B1] Beauducel A. Herzberg P. Y. (2006). On the performance of maximum likelihood versus means and variance adjusted weighted least squares estimation in CFA. *Struct. Equ. Modeling* 13 186–203 10.1207/s15328007sem1302_2

[B2] Bentler P. M. Chou C.-P. (1992). Some new covariance structure model improvement statistics. *Sociol. Methods Res.* 21 259–282 10.1177/0049124192021002006

[B3] Beuckelaer A. Lievens F. Swinnen G. (2007). Measurement equivalence in the conduct of a global organizational survey across countries in six cultural regions. *J. Occup. Organ. Psychol.* 80 575–600 10.1348/096317907X173421

[B4] Bollen K. A. (1989). *Structural Equations with Latent Variables.* New York: John Wiley & Sons, Inc

[B5] Boomsma A. (2013). Reporting Monte Carlo studies in structural equation modeling. *Struct. Equ. Modeling* 20 518–540 10.1080/10705511.2013.797839

[B6] Borsboom D. Romeijn J. W. Wicherts J. M. (2008). Measurement invariance versus selection invariance: is fair selection possible? *Psychol. Methods* 13 75–98 10.1037/1082-989X.13.2.75 18557679

[B7] Byrne B. M. Shavelson R. J. Muthén B. (1989). Testing for the equivalence of factor covariance and mean structures: the issue of partial measurement invariance. *Psychol. Bull.* 105 456–466 10.1037/0033-2909.105.3.456

[B8] Chen F. F. (2008). What happens if we compare chopsticks with forks? The impact of making inappropriate comparisons in cross-cultural research. *J. Pers. Soc. Psychol.* 95 1005–1018 10.1037/a0013193 18954190

[B9] Cheung G. W. Rensvold R. B. (2000). Assessing extreme and acquiescence response sets in cross-cultural research using structural equations modeling. *J. Cross Cult. Psychol.* 31 187–212 10.1177/0022022100031002003

[B10] Davidov E. Meuleman B. Cieciuch J. Schmidt P. Billiet J. (2014). Measurement equivalence in cross-national research. *Annu. Rev. Sociol.* 40 55–75 10.1146/annurev-soc-071913-043137

[B11] DeMars C. E. (2012). A comparison of limited-information and full-information methods in mplus for estimating item response theory parameters for nonnormal populations. *Struct. Equ. Modeling* 19 610–632 10.1080/10705511.2012.713272

[B12] Drasgow F. (1982). Biased test items and differential validity. *Psychol. Bull.* 92 526–531 10.1037/0033-2909.92.2.526

[B13] Drasgow F. (1984). Scrutinizing psychological tests: measurement equivalence and equivalent relations with external variables are the central issues. *Psychol. Bull.* 95 134–135 10.1037/0033-2909.95.1.134

[B14] Flora D. B. Curran P. J. (2004). An empirical evaluation of alternative methods of estimation for confirmatory factor analysis with ordinal data. *Psychol. Methods* 9 466–491 10.1037/1082-989X.9.4.466 15598100 PMC3153362

[B15] Forero C. G. Maydeu-Olivares A. (2009). Estimation of IRT graded models for rating data: limited versus full information methods. *Psychol. Methods* 14 275–299 10.1037/a0015825 19719362

[B16] French B. F. Finch W. H. (2011). Model misspecification and invariance testing using confirmatory factor analytic procedures. *J. Exp. Educ.* 79 404–428 10.1080/00220973.2010.517811

[B17] Furnham A. Guenole N. Levine S. Z. Chamorro-Premuzic T. (2013). The NEO Personality Inventory–Revised: factor structure and gender invariance from exploratory structural equation modeling analyses in a high-stakes setting. *Assessment* 20 14–23 10.1177/1073191112448213 22837539

[B18] Goodman R. (1997). The Strengths and Difficulties Questionnaire: a research note. *J. Child Psychol. Psychiatry* 38 581–586 10.1111/j.1469-7610.1997.tb01545.x 9255702

[B19] Hallquist M. (2011). *MplusAutomation: Automating Mplus Model Estimation and Interpretation.* R package version 0.6. Available at: http://cran.r-project.org/web/packages/MplusAutomation/index.html">

[B20] Holland P. W. Thayer D. T. (1988). “Differential item performance and the Mantel-Haenszel procedure,” in *Test Validity* eds Wainer H. Braum H. (Hillsdale, NJ: Erlbaum) 129–145

[B21] Kankaraš M. Vermunt J. K. Moors G. (2011). Measurement equivalence of ordinal items: a comparison of factor analytic, item response theory, and latent class approaches. *Soc. Methods Res.* 40 279–310 10.1177/0049124111405301

[B22] Kim E. S. Yoon M. (2011). Testing measurement invariance: a comparison of multiple-group categorical CFA and IRT. *Struct. Equ. Modeling* 18 212–228 10.1080/10705511.2011.557337

[B23] Lubke G. H. Dolan C. V. Kelderman H. Mellenbergh G. J. (2003). Weak measurement invariance with respect to unmeasured variables: an implication of strict factorial invariance. *Br. J. Math. Stat. Psychol.* 56 231–248 10.1348/000711003770480020 14633334

[B24] Meade A. W. Lautenschlager G. J. (2004a). A comparison of item response theory and confirmatory factor analytic methodologies for establishing measurement equivalence/invariance. *Organ. Res. Methods* 7 361–388 10.1177/1094428104268027

[B25] Meade A. W. Lautenschlager G. J. (2004b). A monte-carlo study of confirmatory factor analytic tests of measurement equivalence/invariance. *Struct. Equ. Modeling* 11 60–72 10.1207/S15328007SEM1101_5

[B26] Mellenbergh G. J. (1989). Item bias and item response theory. *Int. J. Educ. Res.* 13 127–143 10.1016/0883-0355(89)90002-5

[B27] Meredith W. (1993). Measurement invariance, factor analysis and factorial invariance. *Psychometrika* 58 525–543 10.1007/BF02294825

[B28] Millsap R. E. (1995). Measurement invariance, predictive invariance, and the duality paradox. *Multivariate Behav. Res.* 30 577–605 10.1207/s15327906mbr3004_6 26790049

[B29] Millsap R. E. (1998). Invariance in measurement and prediction: their relationship in the single-factor case. *Psychol. Methods* 2 248–260 10.1037/1082-989X.2.3.248

[B30] Muthén B. O. (2013). *IRT in Mplus.* Available at: http://www.statmodel.com/download/MplusIRT2.pdf">

[B31] Muthén L. K. Muthén B. O. (1998–2011). *Mplus User’s Guide* 6th Edn. Los Angeles, CA: Muthén & Muthén

[B32] Oberski D. L. (2014). Evaluating sensitivity of parameters of interest to measurement invariance in latent variable models. *Polit. Anal.* 22 45–60 10.1093/pan/mpt014

[B33] Paxton P. Curran P. J. Bollen K. A. Kirby J. Chen F. (2001). Monte Carlo experiments: design and implementation. *Struct. Equ. Modeling* 8 287–312 10.1207/S15328007SEM0802_7

[B34] Saris W. E. Satorra A. Sorbom D. (1987). The detection and correction of specification errors in structural equation models. *Soc. Methodol.* 17 105–129 10.2307/271030

[B35] Satorra A. (1989). Alternative test criteria in covariance structure analysis: a unified approach. *Psychometrika* 54 131–151 10.1007/BF02294453

[B36] Schmitt N. Golubovich J. Leong F. T. (2011). Impact of measurement invariance on construct correlations, mean differences, and relations with external correlates an illustrative example using Big Five and RIASEC measures. *Assessment* 18 412–427 10.1177/1073191110373223 20622198

[B37] Schmitt N. Kuljanin G. (2008). Measurement invariance: review of practice and implications. *Hum. Resource Manag. Rev.* 18 210–222 10.1016/j.hrmr.2008.03.003

[B38] Schunn C. D. Wallach D. (2005). “Evaluating goodness-of-fit in comparison of models to data,” in *Psychologie der Kognition: Reden and Vorträge anlässlich der Emeritierung von Werner Tack* ed. Tack W. (Saarbrueken: University of Saarland Press) 115–154

[B39] Stark S. Chernyshenko O. S. Drasgow F. (2006). Detecting DIF with CFA and IRT: toward a unified strategy. *J. Appl. Psychol.* 91 1292–1306 10.1037/0021-9010.91.6.1292 17100485

[B40] Steinmetz H. Schmidt P. Tina-Booh A. Wieczorek S. Schwartz S. H. (2009). Testing measurement invariance using multigroup CFA: differences between educational groups in human values measurement. *Qual. Quant.* 43 599–616 10.1007/s11135-007-9143-x

[B41] Vandenberg R. J. Lance C. E. (2000). A review and synthesis of the measurement invariance literature: suggestions, practices, and recommendations for organizational research. *Organ. Res. Methods* 3 4–70 10.1177/109442810031002

[B42] van de Schoot R. Lugtig P. Hox J. (2012). A checklist for testing measurement invariance. *Eur. J. Dev. Psychol.* 9 486–492 10.1080/17405629.2012.686740

[B43] Wasti S. A. Bergman M. E. Glomb T. M. Drasgow F. (2000). Test of the cross-cultural generalizability of a model of sexual harassment. *J. Appl. Psychol.* 85 766–788 10.1037/0021-9010.85.5.766 11055148

[B44] Wirth R. J. Edwards M. C. (2007). Item factor analysis: current approaches and future directions. *Psychol. Methods* 12 58–79 10.1037/1082-989X.12.1.58 17402812 PMC3162326

[B45] Yang C. Nay S. Hoyle R. H. (2009). Three approaches to using lengthy ordinal scales in structural equation models: parceling, latent scoring, and shortening scales. *Appl. Psychol. Meas.* 34 122–142 10.1177/0146621609338592 20514149 PMC2877522

